# Impulsive alcohol-related risk-behavior and emotional dysregulation among individuals with a serotonin 2B receptor stop codon

**DOI:** 10.1038/tp.2015.170

**Published:** 2015-11-17

**Authors:** R Tikkanen, J Tiihonen, M R Rautiainen, T Paunio, L Bevilacqua, R Panarsky, D Goldman, M Virkkunen

**Affiliations:** 1 Department of Psychiatry, University of Helsinki, Institute of Clinical Medicine, Helsinki, Finland; 2 Research and Development, Rinnekoti Foundation, Espoo, Finland; 3 Department of Clinical Neuroscience, Karolinska Institutet, Stockholm, Sweden; 4 Department of Forensic Psychiatry, University of Eastern Finland, Niuvaniemi Hospital, Kuopio, Finland; 5 National Institute for Health and Welfare, Helsinki, Finland; 6 Department of Psychiatry, Helsinki University Central Hospital, Helsinki, Finland; 7 Department of Psychiatry, New York University School of Medicine, New York, NY, USA; 8 Laboratory of Neurogenetics, National Institute on Alcohol Abuse and Alcoholism, Rockville, MD, USA

## Abstract

A relatively common stop codon (Q20*) was identified in the serotonin 2B receptor gene (*HTR2B*) in a Finnish founder population in 2010 and it was associated with impulsivity. Here we examine the phenotype of *HTR2B* Q20* carriers in a setting comprising 14 heterozygous *HTR2B* Q20* carriers and 156 healthy controls without the *HTR2B* Q20*. The tridimensional personality questionnaire, Brown–Goodwin lifetime aggression scale, the Michigan alcoholism screening test and lifetime drinking history were used to measure personality traits, impulsive and aggressive behavior, both while sober and under the influence of alcohol, and alcohol consumption. Regression analyses showed that among the *HTR2B* Q20* carriers, temperamental traits resembled a passive-dependent personality profile, and the presence of the *HTR2B* Q20* predicted impulsive and aggressive behaviors particularly under the influence of alcohol. Results present examples of how one gene may contribute to personality structure and behaviors in a founder population and how personality may translate into behavior.

## Introduction

Cognitive impulsivity and actual impulsive behaviors show wide inter-individual differences. Impulsivity may enhance performance in some areas of life, but it can also be a trait diagnostic of neuropsychiatric disorders. Several distinct neural pathways contribute to the complex construct of impulsivity.

Bevilacqua *et al.*^1^ discovered a stop codon mutation in the gene encoding for the serotonin 2B receptor (*HTR2B* Q20*), located at 2q36.3-q37.1, in a Finnish founder population. They observed that the stop codon leads to an interruption in the expression of the serotonin 2B (5-HT2B) receptor in lymphoblastoid cells, implying a 50% decrease of the receptor protein's production in heterozygous individuals. The actual function of the 5-HT2B receptor is poorly understood, especially in humans. However, Bevilacqua *et al.*^1^ found that the *HTR2B* Q20* connected with impulsive behavior and cognitive impulsivity. Moreover, they showed that the 5-HT2B receptor is widely expressed in the human brain with the highest densities in the frontal lobe, cerebellum and the occipital lobe, although not all areas of the brain were examined.^1^ The 5-HT2B receptor seems to be required for pharmacological anti-depressive action in mice, which indicates a role of the this receptor in serotonergic neurotransmission.^2^ It has also been shown, in neuronal cells, that 3,4-methylenedioxymethamphetamine (MDMA, commonly known as ‘ecstasy') selectively binds to and activates 5-HT2B receptors, inducing serotonin release in the mouse raphe nuclei, thus leading to dopamine release in the nucleus accumbens and ventral tegmentum.^3^ Moreover, 5-HT2B receptor agonists increase serotonin transporter phosphorylation.^4^

Over 100,000 Finns (2.2%) are carriers of at least 1 *HTR2B* Q20* allele. The phenotype of one homozygote *HTR2B* Q20* male has been described.^1^ He was born prematurely with a low weight, but his development and cognitive capacity were normal. However, he suffered from alcohol dependence already in young adulthood and his drinking caused psychosocial problems, such as a tendency to get into fights while under the influence of alcohol. This finding suggests that the 5-HT2B receptor is not essential for survival in humans.^1^ Conversely, only 50% of *Htr2b* knockout mice survive their first postnatal week, since the 5-HT2B receptor plays a key role in the differentiation of cranial neural crest cells^5^ and heart development.^6^ The 5-HT2B receptor has also been shown to be required to form experimental tumors in nude mice,^7^ and for the development of pulmonary hypertension through bone-marrow contribution,^8^ which suggest a potential preventive role of the *HTR2B* Q20* in some somatic diseases.

The primary focus of interest in our study was on impulsivity, as it is a key feature in many neuropsychiatric disorders.^9^ Intermediate phenotypes, such as impulsivity, may reveal shared biological constructs of diseases that are presently perceived as distinct disorders, as shown for five major psychiatric disorders (schizophrenia, bipolar disorder, major depressive disorder, autism spectrum disorders and attention-deficit/hyperactivity disorder) by the Psychiatric Genomics Consortium.^10^

In addition to the *HTR2B* Q20*, some other preliminary findings of gene involvement in the impulsivity of humans have been reported, such as tryptophan hydroxylase 2 (*TPH2*),^11^ monoamine oxidase A (*MAO-A*),^12^ serotonin 1A receptor (*HTR1A*),^13^ serotonin 1B receptor (*HTR1B*),^14^ serotonin 3B receptor (*HTR3B*),^15^ serotonin transporter (5-*HTT*)^16^ and dopamine transporter (*DAT1, SLC6A4*).^17^ Moreover, preliminary findings suggest genetic involvement in human violent behavior; that is, alleles coding for a low-activity variant of the monoamine oxidase A enzyme (*MAO-A*)^18^ and *CDH13* coding for the T-cadherin protein.^18^

Impulse control is a learned protective mechanism against overt reactions to negative emotions, and also has a genetic foundation.^19^ Preliminary examples of genes affecting emotion include the presynaptic vesicular monoamine transporter 1 (*VMAT1*),^20^ neuropeptide Y (*NPY*),^21^ the Val158Met common functional polymorphism of catechol-*O*-methyltransferase (*COMT*),^22^ *5-HTT*,^23^ variations in the *FKBP5* gene that affect the release of corticotropin-releasing hormone^24^ and a polymorphism in the *PAC1* gene co-operating with the pituitary adenylate cyclase-activating polypeptide.^25^ Despite increasing knowledge of genetic associations with behaviors, strong causal explanations for the heritability of behavior are missing.^26^

Herein, we examine the effects of *HTR2B* Q20* on temperament, impulsive and aggressive behavior both while sober and under the influence of alcohol, and alcohol consumption. One gene rarely explains a large proportion of complex phenotypic traits and human behavior. However, we investigated the possibility of finding at least some effect of the *HTR2B* Q20* on phenotype based on the preliminary work by Bevilacqua *et al.*^1^ The subjects were obtained from a Finnish founder population isolate with a recent bottleneck, which has been estimated to be a reliable group for detection of genetic causal variants with low-frequencies (0.5–5%) in complex disorders.^27^ Founder populations increase the power to detect effects of rare alleles.^1^ Of the 17 Finnish disease alleles, 70–98% of the disease chromosomes are attributable to a single allele.^28^

## Materials and methods

### Participants and groups

Two groups were examined and compared. These two groups were formed from a sample genotyped by Bevilacqua *et al.*^1^ That Finnish cohort comprised violent offenders (*n*=228), their relatives (*n*=352) and controls (295). Subjects of whom we had complete phenotype data available were included in the present study. The violent offenders were excluded because the majority were alcohol dependent and had a personality disorder diagnosis, and thus probably represent a different extreme phenotype than the nonviolent and nonalcoholic subjects included in the present study. The first group comprised carriers of the *HTR2B* Q20* (*n*=14, 57% were males). These subjects were found among the relatives of the violent offenders and healthy controls genotyped without pre-selection for phenotype or genotype. The second group comprised healthy controls without the *HTR2B* Q20* (*n*=156, 100% were males), who were recruited by newspaper ads. The *HTR2B* Q20* carriers were all heterozygotes and comprised seven relatives (86% were females) of the violent offenders and seven males found from the group of healthy controls. The rationale for combining relatives and healthy controls with different genders into one group was to achieve adequate statistical power. Among the seven *HTR2B* Q20* relatives, four were first degree relatives, two were second degree relatives and one was a step-sister. The prevalence of the *HTR2B* Q20* among the relatives was 2.0 and 2.4% among the controls, which matches the allele frequency reported by Bevilacqua *et al.*^1^

There was no difference between the mean age of the groups: 31.2 years (s.d.=10.5) in the *HTR2B* Q20* group and 30.1 years (s.d.=9.4) in the control group. There was no difference in the mean scores of the last grade of obligatory school (scale 4–10), which describes average cognitive performance; 7.4 (s.d.=0.5) in the nonviolent *HTR2B* Q20* group and 7.7 (s.d.=0.8) among controls.

### Mental disorders

All participants were examined with the DSM-III-R semi-structured interview to detect lifetime mental disorders.^29, 30^ Experienced psychiatrists conducted the interviews and two research psychiatrists blind-rated the interview data under the supervision of a senior research psychiatrist. Inter-rater reliability was high, and any differences were resolved by the senior psychiatrist.

### Tridimensional personality questionnaire

The tridimensional personality questionnaire (TPQ) based on the theoretical frames set by Cloninger^31, 32^ was used to measure temperamental traits that are thought to correlate with individual biological differences in neurotransmitters. The TPQ is a self-reporting instrument that is easy to complete within 30 min.

### Brown–Goodwin lifetime aggression scale

Lifetime aggression was measured with the Brown–Goodwin lifetime aggression scale (BGLAS).^33^ A Likert scale was used, with a maximum score of 48 for behavior while sober, and 12 as a maximum while under the influence of alcohol. Impulsive behavior was defined as impulsive sexual events, traveling, moving a residence or changing employer often.

### The Michigan alcohol screening test, lifetime drinking history and mean consumption of alcohol

Behavior related to alcoholism, drinking habits and alcohol consumption were examined with the Michigan alcohol screening test (MAST) and lifetime drinking history (LDH).^34^ The maximum score to measure alcohol-related issues on the MAST Likert scale was 53. The father's drinking was scored separately. The average alcohol consumption was defined as the lifetime total exposure to alcohol, obtained from the LDH divided by the number of drinking years.

### Mean score comparisons

Mean score comparisons were performed to get an overview of the sample and to prepare for the regression analyses. Due to the large quantity of data, we present only the most central results.

### Genetic and molecular analyses

The methodology has been described in detail by Bevilacqua *et al.*^1^ The genetic and molecular analyses were performed at the Laboratory of Neurogenetics, the National Institute on Alcohol Abuse and Alcoholism, the NIH and the Department of Medical Genetics, the University of Helsinki.

### Ethics

Written informed consent was obtained from each participant. The study protocol was approved by the Institutional Review Boards of the University of Helsinki, the Department of Psychiatry and the Helsinki University Central Hospital.

### Statistical analyses

The analysis of variance with Bonferroni correction for multiple comparisons, two-tailed independent sample *t*-test, logistic regression analysis and linear regression analysis were applied. The SPSS 22.0 software (IBM, Armonk, NY, USA) was used and the significance level was set at the 95% confidence interval.

## Results

### Mental disorders and symptoms among the *HTR2B* Q20* carriers

The prevalence of emotional dysregulation, defined as having a histrionic personality disorder or subclinical long-term mood disorders or frequent mood swings, was 78.6%. There was no difference between the relatives of the violent offenders and the stop codon carriers found among the healthy controls (71 vs 85% *t*(12)=0.612, *P*=0.552). There was also no significant gender difference (females 83% vs males 75% *t*(12)=0.350, *P*=0.732). The histrionic personality disorders were only observed among females.

### Personality traits (TPQ), and the impulsive, aggressive, and alcohol-related risk-behaviors (BGLAS and MAST), lifetime mean alcohol consumption and father's drinking (LDH) predictive power to correctly classify the *HTR2B* Q20* carriers from controls

**Figure 1:**
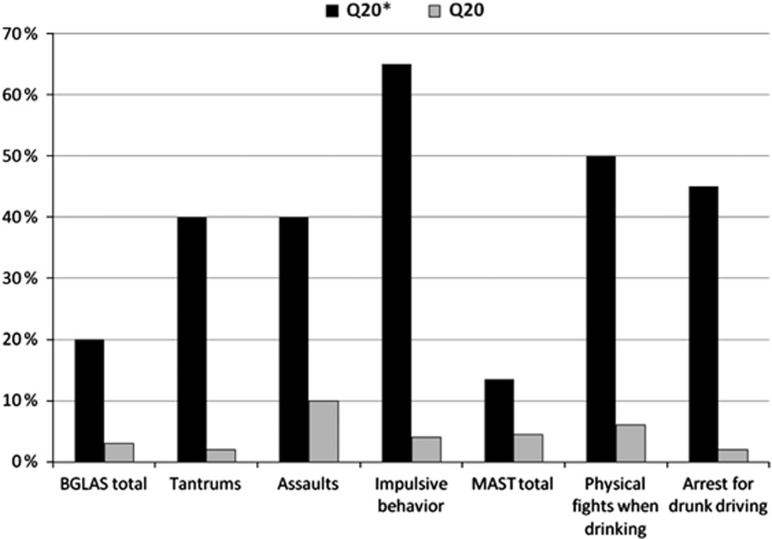
A visualization of high alcohol-related risk-behavior among individuals having a serotonin 2B receptor stop codon (Q20*) and healthy controls (Q20). Brown–Goodwin Lifetime Aggression Scale (BGLAS) scores under the influence of alcohol and Michigan Alcohol Screening Test (MAST) scores have been converted into percentages of the maximum score. All differences are statistically important (*P*<0.05).

The most central results from the bulk of data are presented in Table 1 and Figure 1.

### The relationship between personality traits (TPQ) and the impulsive, aggressive and alcohol-related risk-behaviors (BGLAS and MAST) in the whole sample

Table 2 presents the results in detail. The main trend was that high novelty seeking (NS), high harm avoidance (HA) and high reward dependence (RD) predicted risk-behavior under the influence of alcohol. High NS, high HA and high RD also predicted impulsive and aggressive behaviors while sober, but with smaller effect sizes.

### Control of bias of gender, genetic contamination and environment

Since half (*n*=7) of the *HTR2B* Q20* group consisted of mostly female (six out of seven) relatives to violent offenders, we controlled for a possible divergence of mean values compared with the other half of the *HTR2B* Q20* group comprising only males found among the healthy controls who were not related to the violent offenders. For this analysis of variance with Bonferroni correction for multiple comparisons was applied.

Two differences of mean scores underlying the original results were observed. The *HTR2B* Q20* males had a higher fear of uncertainty (HA2 score) when compared with the nonviolent *HTR2B* Q20* female relatives; 16.3 (s.d.=2.5) vs 7.4 (s.d.=4.5), *P*=0.02, which seems to match the finding of histrionic personality that was only found among females. The *HTR2B* Q20* males also had a higher BGLAS total score; 17.7 (s.d.=6.8) vs 8.6 (s.d.=4.7), *P*=0.006. This finding seems logical, as male sex is thought to be associated with aggression.

Our most important effort to rule out bias caused by genetic contamination, gender and environment was to perform reanalyses of the regression analyses adjusting the analyses with a categorical variable comprising the categories (1) *HTR2B* Q20* relatives to violent offenders (*n*=7), (2) *HTR2B* Q20* controls (*n*=7) and (3) healthy controls (156). The concern was that the relatives could have a considerably different genome as compared with the controls due to that the violent offenders represent an extreme phenotype. Moreover, the relatives were mostly females, which may affect gene expression through epigenetic mechanisms. The relatives could also have experienced challenging environments, as many violent offenders have, that could have induced psychosocial problems. However, adjusting analyses with this categorical variable was the best we could do to control for the possibility of bias. The adjusted reanalyses did not significantly change the results. The adjusted results are presented in the tables.

## Discussion

One of our main findings was that the *HTR2B* Q20* predicted alcohol-related risk-behaviors. The *HTR2B* Q20* carriers demonstrated aggressive out-bursts, got into fights and behaved in an impulsive manner under the influence of alcohol. They were also arrested for driving while under the influence of alcohol more often than the controls. The *HTR2B* Q20* carriers were not alcoholics *per se*, as measured by average alcohol consumption, and were not diagnosed as alcoholics, but they had a tendency to lose behavioral control while under the influence of alcohol.

Another central finding was the high prevalence of mood disorder symptoms and emotional dysregulation among the *HTR2B* Q20* carriers. This was surprising, as the focus of our hypothesis was on impulsivity. However, impulsivity and emotional dysregulation are closely related phenomena. The putative effect of *HTR2B* Q20* on emotional regulation was reported by the study by Diaz *et al.*,^2^ where 5-HTR2B was shown to be required for pharmacological anti-depressive action in mice.

Apart from overt behavior, we observed an effect of the *HTR2B* Q20* on temperament, as a persistent tendency to react to stimuli in a certain way. Though not fully consistent, a pattern matching that of a passive-dependent personality^31^ emerged. Personality features such as relatively low interest in novelty and exploratory activities (low NS total score, NS2 and NS4), anxiety (high HA), fear of uncertainty (high HA2), attachment or dependence (high RD3) and low persistence (low RD2) were characteristic of the *HTR2B* Q20* carriers. Cloninger^31,32^ originally proposed that the personality traits described by the TPQ correlate with underlying neurobiological functions, and, for example, the monoamines serotonin^35,36^ and dopamine^37^ have been shown to play a critical role in human impulsive–aggressive behavior.

Relating results to Cloninger's neurobiological proposal,^31^ the *HTR2B* Q20* carriers may have a low neurophysiological dopaminergic activity. Cloninger associated a high dopaminergic state with impulsivity and high NS.^31^ However, novel neurogenetic research suggests that impulsive decision-making may be associated with low dopamine levels in the prefrontal cortex,^38,39^ which could explain the impulsive behavior of the *HTR2B* Q20* carriers in our sample. Impulsivity caused by high dopaminergic activity is probably a separate construct of impulsive behavior that is mediated primarily by other neuronal pathways.

The high HA finding among the *HTR2B* Q20* carriers is supported by earlier research, as low serotonergic states have been shown to correlate with depression and impulsive–aggressive behavior.^35,36^ Differences found in RD subscales, which are thought to correlate with underlying noradrenergic activity, increase the probability that the *HTR2B* Q20* contributes to neurotransmitter variances, yet the mechanisms are unclear. However, a desychronization in a variety of neuronal networks is thought to cause inhibitory dyscontrol and impaired executive functioning,^40^ which suggests that the observed deviant temperamental features may be linked with alcohol-related impulsive–aggressive behavior and the observed emotional dysregulation.

The acute effects of ethanol on neurotransmission (for example, dopamine release) and behavior may explain these results, in combination with the passive-dependent personality observed in the present study. A causal mechanism would suggest that ethanol enhances an inherent tendency towards impulsive decision-making and subsequently causes acute disinhibition of behavior. Another mechanism could be acute behavioral disinhibition caused by the anxiolytic effects of ethanol since anxiety was high among the *HTR2B* Q20* carriers. Bevilacqua *et al.*^1^ observed an increased motor activity, in *Htr2b* knockout mice, after a D1 receptor agonist challenge (parallel to acute alcohol exposure). Acute ethanol intake could cause a similar fast increase in dopaminergic and motor activity among *HTR2B* Q20* carriers.

We also examined the entire samples' personality trait associations with behavior and found that a passive-aggressive personality^31^ structure (high NS, HA and RD) corresponded with alcohol-related, impulsive–aggressive risk-behavior. Separately, antisocial alcoholic violent offenders have been shown to exhibit an explosive personality^31^ that featured high NS, high HA and low RD.^41^ It seems as if several distinct personality patterns, scoring higher or lower on the scales as compared with controls, are associated with impulsive–aggressive behavior. The *HTR2B* Q20* carriers with a passive-dependent personality may be one distinct subgroup, as the passive-dependent personality has been suggested to be linked with alcohol-related ‘loss of control' behavior.^32^

Even though an impact of the *HTR2B* Q20* on monoaminergic function in the brain would be the most obvious explanation of the results, direct pleiotropic effects of the *HTR2B* Q20* on endocrine function of the body holds explanatory potential since gene–endocrine interactions may alter the risk for impulsivity and alcohol-related problem behavior. For example, an interaction between the functional *MAO-A* polymorphism and testosterone has been shown to alter the risk for antisocial behavior.^42^ Also, the metabolism of glucose and insulin has been shown to directly predict impulsive–aggressive behavior.^43,44,45^ A potential molecular mechanism for this could be that high glycogen synthase kinase 3β (GSK3β) activity downregulates insulin-mediated glycogen synthesis and glucose homeostasis,^46^ but a role for *HTR2B* Q20* in this scenario is partly speculative at this point. The *HTR2B* Q20*, however, may have a role in insulin and glucose metabolism, as the 5-HT2B receptor is required for prolactin-induced β-cell expansion during pregnancy in mice;^47^ *Htr2b* knockout mice remained normo-glycemic after an effort to create a diet-induced, insulin-resistance state.^48^

Despite the fact that one gene rarely explains a large proportion of behaviors, these preliminary results suggest that the *HTR2B* Q20* may have a role in the inter-individual differences of behavior after exposure to alcohol. The study sample belongs to a unique fonder population, which increases the possibility to detect links between genes and behavior. A primary preventive measure against risk-behavior in *HTR2B* Q20* carriers would be to decrease alcohol consumption or achieve abstinence, and increase cognitive control over impulsive behavior and emotions through cognitive psychotherapy, pharmacological treatment and psychosocial interventions.

A potential source of bias of the result was the small sample size, which increases the possibility of spurious results. Sampling, gender bias, genetic contamination and varying environments are also potential sources of bias. The sampling was compromised by the fact that it was not originally designed for this particular study, but on the other hand the sample was genotyped without pre-selection for phenotype or genotype, and the prevalence of the *HTR2B* Q20* equaled that of the general population. The major concern of bias was that half of the *HTR2B* Q20* group comprised mainly female relatives of violent offenders, which could bias results in several ways. The relatives may have had an essentially differing genome from the *HTR2B* Q20* individuals found among the healthy controls due to that the violent offenders represent an extreme phenotype. The female sex of the relatives may also have biased results. Last, the relatives could have shared psychosocially challenging environments and experiences that often occur in the lives of violent offenders. We tried to rule out all these three potentially biasing factors by adjusting the regression analysis with a categorical variable separating the relative *HTR2B* Q20* individuals from the control *HTR2B* Q20* individuals. The reported results are based on this adjustment. Our study setting would need to be replicated in a more homogenous sample. On the other hand, a study setting examining the effect of gender in a mixed sample would be valuable. Our study would also be worth replicating among homozygous individuals, as abnormal phenotypic characteristics are probably more evident among homozygotes. We were not able to control for specific gene–gene or gene–environment interactions specifically, but tried to rule out the potential bias by using a categorical variable in the regression analyses as described above. Nevertheless, we think that these preliminary results contribute towards behavioral genetic understanding of impulsivity and related diseases and risk-behaviors.

## Figures and Tables

**Table 1:** The personality traits and impulsive, aggressive, alcohol-related risk-behavior, alcohol consumption and father's drinking of *HTR2B* Q20* carriers (*n*=14), are presented using multivariate logistic analyses, where healthy controls (*n*=156) were entered into the models as the comparison group

	β *(s.e.)*	W	P	R^2^
*TPQ*				
Novelty seeking total score (NS)	−0.15 (0.17)	4.4	0.036	0.10
Impulsiveness–reflection (NS2)	−0.80 (0.21)	14.8	<0.001	0.28
Disorderliness–regimentation (NS4)	−1.55 (0.79)	3.8	0.048	0.96
Harm avoidance total score (HA)	0.64 (0.14)	20.8	<0.001	0.64
Fear of uncertainty–confidence (HA2)	0.43 (0.15)	8.2	0.004	0.12
Fatigability and asthenia–vigor (HA4)	−0.47 (0.12)	15.8	<0.001	0.45
Persistence–irresoluteness (RD2)	−0.48 (0.12)	15.4	<0.001	0.29
Attachment–detachment (RD3)	0.95 (0.21)	19.9	<0.001	0.50

*BGLAS*				
Total score (S)	0.08 (0.05)	3.3	0.07	0.08
Total score (UIA)	2.28 (0.48)	22.5	<0.001	0.60
Tantrums (S)	0.53 (0.18)	8.4	0.004	0.13
Tantrums (UIA)	4.0 (0.90)	19.6	<0.001	0.32
Assaults (S)	0.48 (0.19)	6.2	0.013	0.11
Assaults (UIA)	3.83 (0.79)	23.4	<0.001	0.35
Impulsive behavior (S)	1.20 (0.30)	16.1	<0.001	0.26
Impulsive behavior (UIA)	4.39 (0.90)	27.8	<0.001	0.43

*MAST*				
Total score	0.48 (0.13)	14.8	<0.001	0.39
Physical fights (UIA)	2.82 (0.65)	19.0	<0.001	0.26
Arrest for driving (UIA)	1.77 (0.40)	19.9	<0.001	0.29

*LDH*				
Alcohol consumption (kg per year)	−0.11 (0.10)	1.13	0.288	0.04
Father's drinking	−0.16 (0.22)	0.52	0.472	0.04

Abbreviations: HA, harm avoidance; NS, novelty seeking; *R*^2^, Nagelkerke *R* square test; RD, reward dependence; S, sober; UIA, under the influence of alcohol; W, Wald's test; β, regression coefficient.

Personality traits were assessed with the tridimensional personality questionnaire (TPQ). Impulsive and aggressive behavior was assessed with the Brown–Goodwin lifetime aggression scale (BGLAS). Alcohol-related risk-behavior was measured with the BGLAS and the Michigan alcohol screening test (MAST). Mean lifetime alcohol consumption and father's drinking were assessed with the Lifetime Drinking History inventory (LDH).

Analyses were adjusted with a categorical variable dividing the sample into three groups: mainly female relative carriers of the *HTR2B* Q20* (*n*=7), male *HTR2B* Q20* carriers found among the healthy controls (*n*=7) and male controls (*n*=156). The rationale for this was to control for gender, genetic contamination and environmental bias.

**Table 2:** The relationship between personality traits and impulsive, aggressive and alcohol-related risk-behavior assessed with linear regression analyses in a sample comprising 156 healthy controls and 14 *HTR2B* Q20* carriers

	β *(s.e.)*	*95% CI*	P	R^*2*^
*Novelty seeking*				
* BGLAS*				
* *Total score (S)	0.13 (0.03)	0.06–0.19	<0.001	0.07
* *Total score (UIA)	2.20 (0.21)	1.79–2.61	<0.001	0.37
* *Tantrums (S)	0.56 (0.21)	0.16–0.96	0.007	0.04
* *Tantrums (UIA)	5.91 (1.02)	3.89–7.92	<0.001	0.14
* *Assaults (S)	0.52 (0.17)	0.18–0.86	0.003	0.05
* *Assaults (UIA)	6.81 (0.83)	5.16–8.45	<0.001	0.26
* *Impulsive behavior (S)	1.36 (0.32)	0.73–1.98	<0.001	0.09
* *Impulsive behavior (UIA)	10.75 (0.88)	9.02–12.47	<0.001	0.44

* MAST*				
* *Total score	0.49 (0.10)	0.30–0.68	<0.001	0.15
* *Physical fights (UIA)	3.57 (0.83)	1.94–5.21	<0.001	0.11
* *Arrest for driving UIA	2.74 (0.54)	1.67–3.81	<0.001	0.14

*Harm avoidance*				
* BGLAS*				
* *Total score (S)	−0.01 (0.04)	−0.09–0.06	0.728	0.01
* *Total score (UIA)	1.32 (0.27)	0.80–1.85	<0.001	0.11
* *Tantrums (S)	0.74 (0.22)	0.30–1.17	0.001	0.06
* *Tantrums (UIA)	5.65 (1.13)	3.43–7.88	<0.001	0.12
* *Assaults (S)	0.23 (0.19)	−0.15–0.60	0.231	0.008
* *Assaults (UIA)	3.61 (1.02)	1.61–5.61	<0.001	0.06
* *Impulsive behavior (S)	0.95 (0.35)	0.26–1.64	0.007	0.04
* *Impulsive behavior (UIA)	8.49 (1.11)	6.30–10.68	<0.001	0.23

* MAST*				
* *Total score	0.43 (0.11)	0.21–0.65	<0.001	0.09
* *Physical fights (UIA)	3.26 (0.95)	1.40–5.13	0.001	0.07
* *Arrest for driving UIA	4.38 (0.55)	3.29–5.47	<0.001	0.29

*Reward dependence*				
* BGLAS*				
* *Total score (S)	0.11 (0.04)	0.03–0.19	0.009	0.04
* *Total score (UIA)	2.71 (0.24)	2.24–3.18	<0.001	0.40
* *Tantrums (S)	0.84 (0.24)	0.37–1.31	0.001	0.06
* *Tantrums (UIA)	9.08 (1.13)	6.84–11.31	<0.001	0.25
* *Assaults (S)	0.54 (0.20)	0.14–0.94	0.009	0.04
* *Assaults (UIA)	7.60 (1.00)	5.62–9.58	<0.001	0.23
* *Impulsive behavior (S)	2.09 (0.36)	1.38–2.80	<0.001	0.15
* *Impulsive behavior (UIA)	14.13 (0.93)	12.29–15.97	<0.001	0.54

* MAST*				
* *Total score	0.77 (0.11)	0.55–0.99	<0.001	0.24
* *Physical fights (UIA)	5.10 (1.00)	3.12–7.07	<0.001	0.15
* *Arrest for driving (UIA)	3.82 (0.65)	2.53–5.11	<0.001	0.18

Abbreviations: CI, confidence interval; *R*^2^, Nagelkerke *R* square test; S, sober; UIA, under the influence of alcohol; β, regression coefficient.

Personality traits are presented as main domains of the tridimensional personality questionnaire (TPQ), and behavior is described with the Brown–Goodwin lifetime aggression scale (BGLAS) and the Michigan alcohol screening test (MAST).
